# Pain and Its Impact on Functional Health: 7-Year Longitudinal Findings among Middle-Aged and Older Adults in Indonesia

**DOI:** 10.3390/geriatrics5020039

**Published:** 2020-06-22

**Authors:** Vasoontara Sbirakos Yiengprugsawan, John Piggott, Firman Witoelar, Fiona M Blyth, Robert G Cumming

**Affiliations:** 1 Australian Research Council Centre of Excellence in Population Ageing Research (CEPAR), Business School, University of New South Wales, Kensington 2033, Australia; j.piggott@unsw.edu.au; 2 Crawford School of Public Policy, The Australian National University, Canberra 2601, Australia; firmanwitoelar.Kartaadipoetra@anu.edu.au; 3 School of Public Health, Faculty of Medicine and Health, The University of Sydney, Sydney 2006, Australia; fiona.blyth@sydney.edu.au (F.M.B.); robert.cumming@sydney.edu.au (R.G.C.); 4 Centre for Education and Research on Ageing (CERA), Concord Repatriation General Hospital, Sydney 2139, Australia

**Keywords:** activities of daily living, aged, chronic pain, functional limitations, persistent pain

## Abstract

Pain is a growing public health issue worldwide, but there is limited population-based evidence in low- and middle-income country settings. Using nationwide Indonesian Family Life Survey (IFLS) data in 2007 and 2014, this research sets out to investigate the associations between changes in pain status between two time points and its impact on functional health outcomes among middle-aged and older adults in Indonesia. Analyses focused on 7936 adults aged 50 years and older in 2014 who responded to both waves. Functional health was assessed using a composite score of functional limitations (range 20–100), representing difficulty in performing activities of daily living, and grip strength (kilograms). Multivariate linear regression models were used to analyse associations between pain measured in 2007 and 2014 and functional health in 2014. Severe pain in the latest wave of IFLS was associated with older age, female, lower education, having chronic conditions or depressive symptoms. Notably, those who reported ‘low–medium’ pain in 2007 and ‘severe’ pain in 2014 belonged to the most vulnerable group with worst functional health outcomes (4.96 points higher limitation scores and 1.17 kg weaker average grip strength). Findings have implications for public health policy in monitoring and management of pain including related co-morbidities as an increasingly critical component of population ageing.

## 1. Introduction

Pain is an increasingly common phenomenon over the lifetime of a person [1,2]. Pain, when persistent, directly impacts the physical and psychological wellbeing of individuals and could have implications for health service use and social support systems [3,4,5]. According to the Global Burden of Disease Study 1990–2017, musculoskeletal disorders comprised the second highest cause of years lived with disability, posing public health challenges in developed and developing countries [6]. These issues may become severe among older people, affecting physical functioning in daily activities, frailty, social engagement, and psychological distress [7,8]. Pain management in older persons is challenging due to other co-morbidities [5]. Chronic pain is reported to predict risks of injurious falls and fractures among the elderly [9,10] and could be linked with an increased risk of cancers, cardiovascular mortality and all-cause mortality [11,12]. 

Internationally, cross-sectional association between pain and functional ability among older adults has been well documented [13,14]. Longitudinal studies, albeit limited, have emerged in recent years, especially on the impacts of multi-site and chronic widespread pain among middle-aged and older adults [10,15,16,17,18]. A 14-year longitudinal study among older respondents in the US reported a 30% increased risk of incident disability and up to 80% increased risk of pain in three or more areas compared with participants who reported no disability at baseline [16]. Longitudinal analyses of data from participants aged 50 years and older in England found that multi-site pain was a strong predictor of limited activities of daily living, and persistent pain over a 6-year period was strongly associated with worse physical performance outcomes [17]. A 4-year longitudinal study among European males found chronic widespread pain to be associated with worsening frailty [18]. A 2-year longitudinal follow-up assessment among men aged 70 years and older in Australia reported incident knee pain was associated with mobility limitations and disability compared with those without knee pain [15].

In Asia, longitudinal data among older adults are mostly from high- or middle–high-income settings. For example, two prospective cohort studies among older Japanese men and women showed that severe pain was independently associated with the incidence of disability ranging from 31% to 66% increase in functional disability [19,20]. Furthermore, a longitudinal study among older Chinese men and women reported that pain is a predictor of worse physical performance after a 4-year follow-up [21], and a 2-year prospective cohort study among older Malaysians reported the longitudinal relationship between chronic pain and elder abuse [22]. Longitudinal evidence is needed to enhance our understanding of the dynamics of pain in middle-aged and later life that is crucial in maintaining functional ability and reducing future care burden.

Indonesia is a lower middle-income Asian country with over 260 million people. It is estimated that the percentage of people aged 60 years and over will reach 12% (34 million) in the next five years and 16% (48 million) in the year 2035 [23]. As of 2019, life expectancy has reached 69 years for males and 74 years for females, and mortality rates have dropped below 200 per 1000 adults over the past two decades [23]. Using nationally representative longitudinal data between 2007 and 2014, we set out to investigate the relationships between pain and its impact on functional health outcomes among middle-age and older adults in Indonesia. The following main research question will add to the limited longitudinal evidence from an Asian emerging economic setting: “To what extent do changes in pain status at the two time points (measured in 2007 and 2014) impact functional health outcomes (2014)?” The study will not only determine the dynamic nature and impact of pain on functional health but also identify population characteristics and co-morbidities associated with pain in later life. Findings could have public health implications applicable to many countries with limited resources facing population ageing challenges. 

## 2. Materials and Methods 

### 2.1. Data

The Indonesian Family Life Survey (IFLS), which began in 1993, is an on-going voluntary longitudinal socioeconomic and health survey with nationally representative samples (83%) that includes 13 of the 27 provinces of the Indonesian population [24]. Within each of the 13 provinces, the representative frame was based on the Indonesian National Socioeconomic Survey (SUSENAS) with 20 households from each urban area and 30 households from each rural area. For IFLS Wave 1 (1993), interviews were conducted with 7224 households [24]. Among the original IFLS Wave 1 household members, one third remains in IFLS 5 (2014). This study is based on the two latest waves of IFLS Wave 4 in 2007 as well as repeated pain and outcome measures at Wave 5 in 2014. There were 19,096 identical respondents in both waves (7936 aged 50 years+). 

The response rate in IFLS Wave 4 in 2007 was 84% of household members at any age and 71% of those aged 50 years and over, compared to 81% at any age and 64% of those aged 50 years and older in IFLS Wave 5 in 2014 [24]. Urban, more highly educated, and higher economic status have lower response rates, and there are indications that over the years, these differences may slightly increase due to migration [24].

IFLS contains comprehensive individual and household characteristics, including age, sex, education, income, household composition, subjective and objective health measures and biomarkers, health risk behaviours, healthcare utilisation, cognitive health and subjective wellbeing. The last two waves of data used in this study were collected by SurveyMETER and RAND Corporation, whereby members from each sampled household completed face-to-face interviews. Objective physical health measurements were carried out by trained interviewers. Sample selection and data collection are available on the RAND website [24]. Questions in the IFLS were harmonised with the international Health and Retirement Study and were translated from English into Bahasa Indonesia and re-translated back into English to confirm that the re-translation agrees with the original question. Questions were verified, piloted, and pre-tested in both urban and rural areas [24].

Data were publicly available and were obtained from RAND Corporation, and the study was approved through the Human Research Ethics Advisory Panel (Protocol HC190554) on 16th July 2019. The IFLS surveys and their procedures were properly reviewed and approved by IRBs (Institutional Review Boards) in the United States (at RAND) and in Indonesia.

### 2.2. Measures

Measures of pain: General pain questions were asked at Wave 4 (2007): “In the last 6 months, were you bothered by pain? How severe is the pain [mild, somewhat painful, severe]?” At the 7-year follow-up (2014), respondents were asked “yesterday, did you feel pain [no/a little, somewhat/quite a bit, very much]?” Measures of pain between the two waves were classified using information on persistence (whether pain occurred in both waves) and severity (low, medium, severe). 

In addition, the 2014 survey includes a separate question on pain sites: “Yesterday, were you bothered by a pain in your…? [yes, very much; yes, to some degree; no, not much]” The specific locations include head, neck/shoulder, arm, hand/wrist/finger, back/lower back, hip, knee, ankle/foot/toe, or leg. 

Limitations in activities of daily living: At both Waves 4 (2007) and 5 (2014), subjective physical functioning measures included 14 items of Activities of Daily Living (ADL) and Instrumental Activities of Daily Living (IADL), e.g., walk 1–5 metres, to dress without help, to bathe, to shop, to carry a heavy load. Each question was asked by an interviewer (no measurement involved) [24]. Difficulty levels for each measure ranged from ‘easily’ (score 1) to ‘with some difficulty’ (score 3) and ‘unable to do’ (score 5). Scores were aggregated and converted from the original scale of 14–70 into the scale of 20–100. Higher composite scores of functional limitations represent more difficulty in performing activities of daily living. 

Objective physical performance was measured by average grip strength in kilograms using a Baseline Smedley Spring type dynamometer [24]. The interviewers, many of whom were recent graduates recruited from the school of public health or the nursing academy, were trained in taking physical measurements of health and asked the respondent to squeeze the dynamometer in each hand. Grip strength was taken two times for each hand; the four attempts were then averaged. Grip strength was used as a predictor of old age disability and a biomarker for functional ability in later life [25]. The lower the average score (in kilograms), the weaker the grip strength, which indicates poorer objective health status and frailty.

Covariates: Analyses took into account demographic characteristics (age, sex), socioeconomic status (tertiles of per capita expenditure, education levels) and geographical locations (urban–rural and provinces). Health covariates included baseline functional health (as reported 2007) and co-morbidity of other chronic conditions (whether currently taking medication on a weekly basis for any of these conditions: tuberculosis and other lung conditions, heart problems, stroke, cancer, arthritis, kidney diseases and digestive conditions). In addition, mental health states, especially depression, were found to mediate the relationship between pain and frailty among older persons [26]. IFLS includes the international standardised Center for Epidemiologic Studies Depression Scale (CES-D: 10 items total scores range from 0 to 30) to assess depression with scores of 10 or more classified as having depressive symptoms [27]. Multicollinearity between covariates was tested, and the highest correlation coefficients between the covariates were 0.43 between age and marital status and 0.25 for education and per capita expenditure. The variance inflation factor was less than 5.

### 2.3. Statistical Analysis

Descriptive analyses of pain by socio-demographic status was reported along with chi-square and *p* values for respondents aged 50 years and over. The adopted alpha value for the study is *p* < 0.05. Multivariate linear regression models were used to analyse associations between pain status reported in 2007 and 2014 and functional health outcomes in 2014 among middle-aged and older adults. Hierarchical linear regression approaches were used for analyses. *Model 1* includes sociodemographic variables, *Model 2* includes *Model 1* and health covariates, and *Model 3* includes *Model 2* and pain-related variables. Model summary statistics (adjusted R square and R square change) are included for each outcome—functional limitations and grip strength. Population weights were applied to descriptive proportions as well as in the adjusted linear coefficients. Final analyses excluded those with severe functional limitations (at baseline) in 2007 and were adjusted for sociodemographic attributes and health covariates in 2014.

## 3. Results

Sociodemographic characteristics of study respondents are reported in Table 1. Middle-aged and older adults of interest in this study were aged 50 years and older; about 22.5% were aged 50–59 years, 14.3% were aged 60–69 years and 9.4% were aged 70 years or over. 

Severe pain in the latest wave of IFLS (2014) was more common among females (12.3% compared to 7.7% across all age groups and 15.6% vs. 9.7% among middle-aged and older adults) (Table 1). Respondents who were widowed, had up to elementary school education and belonged to the lowest per capita expenditure tertile reported chronic conditions (that required weekly medication), and those with depressive symptoms had a higher proportion of severe pain both across all ages and among those aged 50 years and older. Between 2007 and 2014, crude functional limitation scores tended to increase, and grip strength declined in the crude population; weighted data had a higher proportion of severe pain both across all ages and those aged 50 years and over. 

Characteristics of those with and without severe pain among older adults were all different, which was statistically significant for sex, age groups, marital status, education, and provinces (*p* < 0.05) but not for tertile per capita expenditure (*p* = 0.228) and urban–rural areas (*p* = 0.092). Health attributes and functional health were also statistically different between those aged 50 years and over with and without severe pain (Table 1).

Specific pain sites were recorded separately in the survey which included selected upper and lower body parts (Table 2). Notable patterns of specific pain sites include a higher proportion of lower body pain among middle-aged and older adults than the younger group; for example, legs (about 25% compared to 15%), hip (highest among older males), and knees (highest among older females). Multi-site pain was more commonly reported among females in both age groups (12.9% vs. 17.9% and 22.1% vs. 26.6%, respectively). There was a notable statistically significant difference in head, hand/wrist/finger, back/lower back, and leg pain (*p* < 0.001) among males and females in both the younger and older age groups (Table 2). Females reported higher knee pain than males in both age groups and these differences were statistically significant among older groups (*p* < 0.001) but not for the younger one (*p* = 0.070). Pain in upper (neck/shoulder and arm) and lower (ankle/foot/toe) limbs was not statistically different between males and females in either age groups. Reported multi-site pain was statistically significantly higher among females than males (17.9% vs. 12.9% in the younger group and 26.6% vs. 22.1% among the older group).

To investigate pain status at the two waves (2007 and 2014), the overall pain questions were used to create five broad pain status categories among middle-aged or older respondents. There were 18.4% (*n* = 1378) who reported ‘low’ levels of pain in both periods, 6.9% (*n* = 556) who reported ‘severe’ pain in 2007 and ‘low–medium’ pain in 2014, 11.3% (*n* = 954) who reported ‘low–medium’ pain in 2007 and ‘severe’ pain in 2014 and 1.9% (*n* = 156) who reported severe ‘pain’ in both periods (Table 3). 

Hierarchical linear regression approaches were used to investigate associations between changes in pain status between two time points and its impact on functional health outcomes. *Model 1* includes sociodemographic variables, *Model 2* includes *Model 1* and health covariates and *Model 3* includes *Model 2* and pain-related variables (Table 3). The effects of age, sex, being a widow, having elementary education, and residing in outer islands were strongly significant across the three models. Depression scores and chronic conditions were strongly associated with functional limitation scores in both models. 

To test cross-sectional associations between pain and functional outcomes in 2014, respondents aged 50 years and older who reported ‘severe’ pain had 6.59 points higher functional limitation scores and 1.36 points weaker grip strength compared to those reporting ‘no pain’ (data not shown, available as a Supplementary File). 

In the longitudinal analyses, multivariable linear regression results in *Model 3* revealed that those reporting ‘low–medium’ pain in 2007 and ‘severe’ pain in 2014 were the group with worst functional limitation scores followed by those with ‘severe’ pain in both 2007 and 2014 (4.96 points and 3.91 points higher functional limitation scores, respectively) (Table 3). Notably, having multi-site pain was associated with 0.91 higher limitation scores, but this was not statistically significant. Residents of outer islands had 2.04 points higher functional limitation scores. Respondents with elementary school education and widowers were associated with 1.76 and 1.38 points higher functional limitation scores. In the fully adjusted analyses, having depressive symptoms and chronic conditions was associated with 4.38 and 2.05 points higher functional limitation scores, respectively. 

Relationships between pain status and an objective measure of physical performance (grip strength) are reported in Table 4. Being female; of older age; being separated, divorced or widowed and having lowest per capita expenditure was significantly associated with weaker grip strength in all three models. Depression scores and chronic conditions were strongly associated with weaker grip strength in both models. In the fully adjusted Model 3, reporting ‘low–medium’ pain in 2007 and ‘severe’ pain in 2014 was associated with 1.17 points weaker grip strength. Having depressive symptoms based on the CES-D scale and chronic conditions was associated with 0.91 and 0.67 points higher limitation scores, respectively. Changes in R-square in *Models 2* and *3* were statistically significant for both functional health outcomes (*p* < 0.05). Notably, greater variance of self-reported functional limitation could be due to the composite nature of the scores which covered the whole body (derived from 14 ADL and IADL items, see Methods section), in contrast to the objective single grip strength measure.

## 4. Discussion 

This study contributes to existing but limited longitudinal evidence on the causal links between pain and functional health among older community dwelling adults in low- and middle-income Asian countries. In particular, we found that respondents aged 50 years and over who reported ‘low–medium’ levels of pain in 2007 and ‘severe’ pain in 2014 were strongly associated with both functional limitation scores and weaker grip strength followed by those who reported ‘severe’ pain in both 2007 and 2014, after adjusting for sociodemographic characteristics and health covariates. We note the significant impact of ‘low–medium’ pain in 2007 and ‘severe’ pain in 2014, which could be more recent, and the older respondents might have had less time to adjust to these functional limitations and its impact on physical performance than those who already had ‘persistent’ pain (severe in both periods).

Among demographic covariates, female, older age groups and being widowed was related to both pain and adverse functional health. Notably, a study has noted that an association between pain intensity and disability was more attenuated in older compared to younger age groups [14]; however, pain among older persons is often chronic and related to multiple morbidities which further contribute to functional limitation in later life [2,5]”. Among socioeconomic covariates, those with low education were strongly associated with reporting pain and adverse functional health. Low levels of education could limit health literacy, nutrition, and access to health care disadvantaging health in later life [28]. The findings also point out the need to consider co-morbidities such as chronic conditions on physical health, but also psychological health (e.g., depressive symptoms) when investigating pain and functional health [26,29].

Our longitudinal findings complement other cross-sectional studies in Indonesia which provide insight into the impacts of pain on quality of life and healthcare seeking behaviours in Jakarta and East Java [30,31]. A longitudinal study in Thailand found that persistent and incident lower back pain was associated with functional limitations over a 4-year period [32], and a prospective study of pain among Iranians found incident neck and shoulder pain among office workers and subsequent impacts during the 12-month follow-up period [33]. Chronic pain through middle-aged years could have medium- and long-term health implications [2,3]. Our empirical findings among community-dwelling older persons in Indonesia further confirmed such impacts of pain on daily activities in later life. In emerging economies in Southeast Asia, recent collaborative developments in this area include pilot multidisciplinary pain clinics with local partners in Indonesia, Myanmar and Vietnam [34].

Population-based longitudinal surveys such as IFLS have the advantage of documenting self-reported pain and its impact over time. It is important to caution some study limitations; firstly, attribution in IFLS was primarily documented to be from loss of follow-up from movement out of areas, and these non-respondents tended to be of a higher socioeconomic status [24,35]. Because pain and functional health were linked in our study to low socioeconomic status, this attribution bias is less likely to affect the overall findings. Secondly, the two functional health outcomes in the study were not assessed for validity and reliability in the Indonesian setting; however, these measures are part of international standardised Health and Retirement Study, and both activities of daily living and grip strength were recommended for research and care practice in older persons [25,36]. Thirdly, the self-reported general pain in the two waves of IFLS questionnaires does not reflect its acute or chronic nature. Our results therefore could not distinguish between causes of pain, including disability, and related psychological factors such as pain catastrophising and pain self-efficacy. Pain among older persons is complex, and it is often difficult to distinguish between pain related to nociceptive processing in (multiple) peripheral sites and pain arising from disturbances to the central nervous system [37,38]. Lastly, another limitation is that pain was measured at two time points 7 years apart (2007 and 2014); hence, it was not possible to capture pain that may have occurred in between the survey periods. However, the use of an overall pain question in our longitudinal analyses has the advantage of capturing severity of pain at the two time points irrespective of the causes.

Levels of pain used in this study were self-reported, which is commonly applied in international nationwide surveys [10,17]. However, we acknowledge that individuals may have varying thresholds of pain subject to their biological and socio-cultural environment [39]. Interpretation of our findings should take into account the inter-link between ageing, physical decline, and pain in cross-cultural settings [40]. Moreover, approaching pain research from a multidisciplinary approach through qualitative data is crucial in shedding light on age-related perceptions and experiences living with chronic pain and may help minimise barriers in seeking health care and management of pain among older persons [5].

## 5. Conclusions

The study findings on the longitudinal impacts of pain on functional heath on older persons highlight the importance of monitoring pain and other co-morbidities of chronic conditions and depressive symptoms. Severe pain which leads to functional limitations and loss of independence could increase future demand for care and health services and require social support interventions. In an increasingly ageing society, population surveys could be a helpful tool to raise awareness for screening and early public health interventions. Better management of pain could contribute to maintaining a healthy ageing lifestyle and independence, which will be crucial in later life.

## Figures and Tables

**Table 1:** Sample characteristics by pain level, Indonesian Family Life Survey (IFLS) 2014.

Attributes (*n* = 19,096)	Population Weighted %		Reporting Severe Pain (n)		Characteristics by Pain Status, Age 50+		Chi-Square ^†^
	All Age	50+ Years	All Age	50+ Years	Without Severe Pain	With Severe Pain	*t*-test ^‡^
Overall				13.2 (1110)			*p* < 0.001 ^†^
Male	41.9	40.3	7.7 (574)	9.7 (286)	41.9	29.4	
Female	58.1	59.7	12.3 (1407)	15.6 (823)	58.0	70.6	
*Age groups*							*p* < 0.001 ^†^
<35	19.2		6.9 (331)				
35–49	34.5		8.6 (541)				
50–59	22.5	48.7		11.1 (426)	49.9	41.0	
60–69	14.3	31.0		13.7 (327)	30.8	32.2	
70+	9.4	20.4		17.4 (356)	19.4	26.8	
*Marital status*							*p* < 0.001 ^†^
Married	77.9	68.1	9.3 (1361)	9.6(616)	69.7	57.7	
Not married	4.7	0.9	6.6 (78)	11.2 (4)	0.9	0.6	
Separated/divorced	4.0	4.4	14.6 (118)	15.0 (63)	4.3	5.0	
Widowed	13.3	26.7	17.2 (439)	18.2 (426)	25.1	36.6	
*Education*							*p* < 0.001 ^†^
Up to elementary school	9.6	16.8	15.8 (275)	15.9 (234)	16.3	20.3	
Elementary school	45.1	59.4	12.9 (1057)	14.8 (712)	58.3	66.9	
Junior to high school	36.0	18.8	7.1 (537)	8.0 (132)	20.0	11.4	
College/university	8.3	4.9	4.6 (98)	4.1 (17)	5.5	1.5	
*Per capita expenditure*							*p* = 0.228 ^†^
Tertile 1 (lowest)	35.5	37.6	11.0 (697)	14.3 (375)	37.8	36.1	
Tertile 2 (middle)	33.0	31.9	10.4 (658)	13.2 (325)	31.6	33.7	
Tertile 3 (highest)	31.5	30.5	9.8 (621)	12.6 (373)	30.6	30.2	
*Geographical areas*							*p* = 0.092 ^†^
Urban	52.3	52.0	10.6 (1194)	13.9 (614)	51.8	54.4	
Rural	47.7	48.0	10.2 (782)	12.6 (459)	48.2	45.6	
*Provinces*							*p* = 0.025 ^†^
All Sumatra provinces	14.7	14.0	11.5 (490)	14.2 (253)	13.8	15.1	
Jakarta	5.7	5.6	13.3 (159)	16.6 (80)	5.4	7.1	
The rest of Java	66.6	67.7	10.1 (871)	12.8 (497)	68.1	65.7	
Outer islands and other	12.9	12.7	9.5 (462)	12.7 (280)	12.7	12.1	
Health attributes							
Chronic conditions *	9.0	12.6	17.6 (298)	19.7 (213)	11.6	18.7	*p* < 0.001 ^†^
Depressive symptoms (CES-D, scores ≥ 10)	26.1	23.4	32.5 (759)	45.9 (356)	20.5	45.9	*p* < 0.001 ^†^
Depressive symptoms (score 0–30), mean [sd]	6.7 [5.1]	6.4 [4.9]	9.7 [6.0]	9.6 [5.7]	6.5 [4.8]	9.6 [5.7]	*p* < 0.001 ^‡^
2007 Functional limitation (score 20–100), mean [sd]	24.1 [7.8]	25.6 [9.1]	26.9 [11.6]	28.4 [12.7]	25.0 [8.2]	28.4 [12.7]	*p* < 0.001 ^‡^
2014 Functional limitation (score 20–100), mean [sd]	30.3 [11.6]	34.0 [14.3]	37.8 [17.3]	42.8 [18.9]	32.3 [12.6]	42.8 [18.9]	*p* < 0.001 ^‡^
2007 Grip strength, mean in kg [sd]	25.8 [10.6]	23.3 [10.3]	23.6 [9.7]	20.9 [8.7]	21.9 [9.9]	20.9 [8.7]	*p* < 0.001 ^‡^
2014 Grip strength, mean in kg [sd]	24.7 [9.0]	21.2 [8.0]	21.5 [8.7]	18.9 [7.5]	21.5 [8.0]	18.9 [7.5]	*p* < 0.001 ^‡^

* Percentage who reported taking medication on a weekly basis in 2014 for at least one of these chronic conditions: tuberculosis and other lung conditions, heart problems, stroke, cancer, arthritis, kidney diseases, and digestive conditions. ^†^ chi square; ^‡^ *t*-test.

**Table 2:** Pain sites among those reported pain, IFLS 2014.

Specific-Site and Multi-Site Pain	<50 Years		Chi-Square	50+ Years		Chi-Square
	Male	Female		Male	Female	
Head	23.2	26.6	*p* < 0.001	9.3	13.2	*p* < 0.001
Neck/shoulder	14.6	13.6	*p* = 0.153	17.8	7.4	*p* < 0.362
Arm	2.8	2.7	*p* = 0.299	4.6	3.4	*p* = 0.282
Hand/wrist/finger	10.8	12.3	*p* < 0.001	10.9	5.2	*p* < 0.001
Back/lower back	24.0	17.5	*p* < 0.001	12.5	22.7	*p* < 0.001
Hip	2.0	3.9	*p* = 0.125	7.2	4.5	*p* = 0.090
Knee	2.9	5.4	*p* < 0.070	5.9	10.9	*p* < 0.001
Ankle/foot/toe	4.0	2.4	*p* = 0.376	5.9	5.1	*p* = 0.275
Leg	14.5	15.1	*p* < 0.001	23.9	25.5	*p* < 0.001
Multi-site	12.9	17.9	*p* < 0.001	22.1	26.6	*p* < 0.001

**Table 3:** Pain status (2007 and 2014) and functional limitation scores (2014), IFLS 2007 and 2014.

Respondents N = 7936 Aged 50 Years+	Functional Limitation Scores ^1^		
	Linear Regression Coefficients [95% Confidence Interval]		
	*Model 1*	*Model 2*	*Model 3*
**Sociodemographic covariates**			
Female (reference: male)	**1.06 [0.69; 1.44]**	**1.18 [0.82; 1.54]**	**0.66 [0.29; 1.03]**
Age (reference: 50–59 years)			
60–69 years	**2.67 [1.98; 3.37]**	**2.43 [1.76; 3.09]**	**1.73 [1.08; 2.37]**
70+ years	**13.2 [11.9; 14.4]**	**12.3 [11.1; 13.5]**	**10.5 [9.24; 11.7]**
Married (reference)			
Not married	−1.39 [3.39; 0.61]	−1.78 [−4.10; 0.56]	−1.27 [−3.23; 0.68]
Separated/divorced	0.18 [−1.29; 1.65]	−0.37 [−1.78; 1.02]	−0.10 [−1.45; 1.24]
Widowed	**2.14 [1.19; 3.09]**	**1.81 [0.89; 2.74]**	**1.38** [0.48; 2.28]
Per capita expenditure: tertile 1 (lowest)	0.74 [−0.18; 1.60]	**1.04 [0.17; 1.90]**	0.78 [−0.04; 1.61]
Per capita expenditure: tertile 2	−0.10 [−0.88; 0.86]	0.31 [−0.53; 1.16]	0.26 [−0.56; 1.07]
Per capita expenditure: tertile 3 (reference)			
Education: Elementary school	**3.07 [1.82; 4.33]**	**1.93 [0.51; 3.30]**	**1.76 [0.45; 3.08]**
Education: Junior to high school	**3.02 [1.64; 4.42]**	**1.73 [0.51; 1.96]**	**1.10 [−0.09; 2.30]**
Education: College (reference)			
Jakarta	**0.97 [0.19; 1.76]**	**1.28 [0.51; 2.06]**	−0.16 [−0.94; 0.62]
The rest of Java	−0.38 [1.62; 0.85]	−0.18 [−1.36; 1.00]	**−1.13 [−2.24; −0.02]**
Outer islands and other	**3.43 [2.60; 4.26]**	**3.14 [2.32; 3.95]**	**2.04 [1.25; 2.83]**
Sumatra province (reference)			
Rural (reference: urban)	−0.33 [−1.10; 0.43]	0.02 [−0.73; 0.77]	0.33 [−0.39; 1.05]
**Health covariates** ^2^			
Depression score (CES-D ≥ 10) ^3^		**5.53 [4.74; 6.33]**	**4.38 [3.59, 5.17]**
Chronic conditions in 2014 ^4^		**2.52 [1.48; 3.57]**	**2.05 [1.03; 3.06]**
**Pain status**			
**2007 and 2014 reported pain level** ^5^			
2007 low|2014 low (reference)			
2007 low–medium|2014 low–medium			−0.60 [−1.71; 0.49]
2007 severe|2014 low–medium			−0.61 [−2−03; 0.82]
2007 low–medium|2014 severe			**4.96 [3.31; 6.62]**
2007 severe|2014 severe			**3.91 [0.89; 6.97]**
**Number of pain sites reported in 2014**			
No pain site (reference)			
Single site			0.48 [−0.45; 1.41]
Multi-site			0.91 [−0.11; 1.93]
*Model adjusted R^2^ (∆ R^2^)*	*0.16*	0.25 (0.09 *)	0.32 (0.06 *)

^1^*Model 1* includes sociodemographic variables; *Model 2* includes *Model 1* + health covariates; *Model 3* includes *Model 2* and pain variables. Bolded values were statistically significant at *p* < 0.05. ^2^ Final analyses excluded those with severe functional limitations (at baseline) in 2007 and adjusted for baseline scores. ^3^ Based on the Center for Epidemiologic Studies Depression Scale (CES-D), scores of ≥10 classified as having depressive symptoms. ^4^ Respondents who reported taking medication on a weekly basis in 2014 for at least one of these chronic conditions: tuberculosis and other lung conditions, heart problems, stroke, cancer, arthritis, kidney diseases and digestive conditions. ^5^ Overall pain questions were asked in 2007 and at the 7-year follow-up (2014). Pain status was classified by persistence (whether pain occurred in both waves) and severity (low, medium, severe). * *∆ R^2^* *p* < 0.05.

**Table 4:** Pain status (2007 and 2014) and grip strength (2014), IFLS 2007 and 2014.

Respondents N = 7936 Aged 50 Years+	Grip Strength, Kilograms ^1^		
	Linear Regression Coefficients [95% Confidence Interval]		
	*Model 1*	*Model 2*	*Model 3*
**Sociodemographic Covariates**			
Female (reference: male)	**−4.91 [−5.07; −4.73]**	**−3.63 [−3.83; −3.47]**	**−3.61 [−3.80; −3.42]**
Age (reference: 50–59 years)			
60–69 years	**−3.04 [−3.39; −2.69]**	**−2.32 [−2.65; 1.99]**	**−2.28 [−2.61; −1.95]**
70+ years	**−6.59 [−7.04; −6.14]**	**−5.18 [−5.62; −4.74]**	**−5.15 [−5.59; −4.70]**
Married (reference)			
Not married	−0.53 [−1.66; 0.61]	0.09 [−1.09; 1.26]	0.07 [−1.15; 1.29]
Separated/divorced	**−1.24 [−1.97; −0.51]**	**−1.05 [−1.72; −0.38]**	**−1.03 [−1.72; −0.35]**
Widowed	**−1.02 [−1.39; −0.66]**	**−0.85 [−1.19; −0.51]**	**−0.85 [−1.19; −0.50]**
Per capita expenditure: tertile 1 (lowest)	**−1.42 [−1.81; −1.03]**	**−0.93 [−1.29; −0.56]**	**−0.96 [−1.33; −0.59]**
Per capita expenditure: tertile 2	−0.25 [−0.63; 0.13]	−0.16 [−0.51; 0.18]	−0.18 [−0.52; 0.16]
Per capita expenditure: tertile 3 (reference)			
Education: Elementary school	−0.52 [−1.21; 0.17]	−0.53 [−1.20–0.15]	−0.42 [−1.09; 0.26]
Education: Junior to high school	0.18 [−0.55; 0.90]	−0.02 [−0.72; 0.68]	0.05 [−0.65, 0.75]
Education: College (reference)			
Jakarta	0.24 [−0.15; 0.64]	**−0.41 [−0.77; −0.04]**	**−0.39 [−0.78, −0.03]**
The rest of Java	−0.31 [−0.90; 0.28]	**−0.90 [−1.44; −0.36]**	**−0.89 [−1.43; −0.34]**
Outer islands and other	−0.09 [−0.43; 0.23]	0.01 [−0.31; 0.32]	−0.01 [−0.32; 0.31]
Sumatra province (reference)			
Rural (reference: urban)	−0.32 [−0.64; 0.01]	−0.08 [−0.39; 0.22]	−0.11 [−0.41; 0.19]
**Health covariates** ^2^			
Depression score (CES-D ≥ 10) ^3^		**−0.86 [−1.17; −0.56]**	**−0.91 [−1.36; −0.46]**
Chronic conditions in 2014 ^4^		**−1.01 [−1.46; −0.57]**	**−0.67 [−0.98; −0.35]**
**Pain status**			
**2007 and 2014 reported pain level** ^5^			
2007 low|2014 low (reference)			
2007 low–medium|2014 low–medium			−0.15 [−0.61; 0.30]
2007 severe|2014 low–medium			0.10 [−0.59; 0.79]
2007 low–medium|2014 severe			**−1.17 [−1.88; −0.47]**
2007 severe|2014 severe			−0.39 [−1.49; 0.71]
**Number of pain sites reported in 2014**			
No pain site (reference)			
Single site			0.14 [−0.25; 0.53]
Multi-site			−0.41 [−0.86; 0.30]
*Model adjusted R^2^ (∆ R^2^)*	*0.56*	0.57 (0.01 *)	0.59 (0.05 *)

^1^*Model 1* includes sociodemographic variables; *Model 2* includes *Model 1* + health covariates; *Model 3* includes *Model 2* and pain variables. Bolded values were statistically significant at *p* < 0.05. ^2^ Final analyses excluded those with severe functional limitations (at baseline) in 2007 and were adjusted for baseline scores. ^3^ Based on the Center for Epidemiologic Studies Depression Scale (CES-D), scores of ≥10 classified as having depressive symptoms. ^4^ Respondents who reported taking medication on a weekly basis in 2014 for at least one of these chronic conditions: tuberculosis and other lung conditions, heart problems, stroke, cancer, arthritis, kidney diseases, and digestive conditions. ^5^ Overall pain questions were asked in 2007 and at the 7-year follow-up (2014). Pain status was classified by persistence (whether pain occurred in both waves) and severity (low, medium, severe). * *∆ R^2^* *p* < 0.05.

## Supplementary Materials

The following are available online at https://www.mdpi.com/2308-3417/5/2/39/s1, Table S1: Cross-sectional associations between pain status and functional health, IFLS 2014.
