# Supplementary material for: Pain and Its Impact on Functional Health: 7-Year Longitudinal Findings among Middle-Aged and Older Adults in Indonesia
Source: Geriatrics (Basel). 2020 Jun 22;5(2):39. doi: 10.3390/geriatrics5020039 (PMC7345182; doi:10.3390/geriatrics5020039)
Supplement: Supplementary file 1 [file geriatrics-05-00039-s001.pdf]

Supplementary file S1: Cross-sectional associations between pain status and functional health, IFLS 2014

Respondents N=7936 aged 50 years+	Linear regression coefficients [95% Confidence Interval][1]	
	Functional limitation scores	Grip strength
**Reported pain level in 2014**		
No pain (reference)		
A little	0.43 [-0.52; 1.39]	-0.35 [-0.82; 0.12]
Quite a bit	**1.31 [0.25; 2.36]**	**-0.59 [-1.04; -0.14]**
Severe	**6.59 [5.25; 7.93]**	**-1.36 [-1.94; -0.79]**
**Sociodemographic covariates**		
Female (reference: male)	**1.08 [0.72; 1.44]**	**-4.88 [-5.05; -4.71]**
Age (reference: 50-59 years)		
60-69 years	**2.30 [1.65; 2.95]**	**-2.95 [-3.30; -2.61]**
70+ years	**12.3 [11.2; 13.5]**	**-6.42 [-6.87; -5.96]**
Married (reference)		
Not married	-0.94 [-3.20; 1.31]	-0.45 [-1.66; 0.76]
Separated/ divorced	-0.38 [-1.74; 0.97]	**-1.09 [-1.82; -0.35]**
Widowed	**1.69 [0.77; 2.61]**	**-0.97 [-1.34; -0.61]**
Per capita expenditure: tertile 1 (lowest)	0.52 [-0.30; 1.33]	**-1.50 [-1.89; -1.11]**
Per capita expenditure: tertile 2	1.10 [0.25; 1.94]	-0.30 [-0.67; 0.07]
Per capita expenditure: tertile 3 (reference)		
Education: Elementary school	**1.39 [-0.14; 2.32]**	-0.21 [-0.92; 0.50]
Education: Junior to high school	**1.64 [0.28; 3.01]**	0.04 [-0.67, 0.07]
Education: College (reference)		
Jakarta	1.04 [0.27; 1.81]	**0.21 [-0.18, 0.60]**
The rest of Java	-0.06 [-1.19; 1.08]	**-0.21 [-0.79; 0.37]**
Outer islands and other	**3.28 [2.48; 4.08]**	-0.08 [-0.41; 0.26]
Sumatra province (reference)		
Rural (reference: urban)	0.31 [-0.44; 1.05]	**-0.38** [-0.70; -0.06]
**Health covariates**[2]		
Depression score (CES-D ≥10)[3]	**4.58 [3.78, 5.37]**	**-0.83 [-1.29; -0.36]**
Chronic conditions in 2014[3]	**1.97 [0.95; 3.00]**	**-0.85 [-1.19; -0.52]**
Model adjusted R^2	*0.26*	*0.54*

[1]Final model includes sociodemographic variables, health covariates, and pain variables. Bolded values were statistically significant at $p<0.05$.

[2]Based on the Center for Epidemiologic Studies Depression Scale (CES-D), scores of ≥10 classified as having depressive symptoms.

[3]Respondents who reported taking medication on a weekly basis in 2014 for at least one of these chronic conditions: tuberculosis and other lung conditions, heart problems, stroke, cancer, arthritis, kidney diseases, and digestive conditions.
